# The One Health Consortium: Design of a Phase I Clinical Trial to Evaluate M032, a Genetically Engineered HSV-1 Expressing IL-12, in Combination With a Checkpoint Inhibitor in Canine Patients With Sporadic High Grade Gliomas

**DOI:** 10.3389/fsurg.2020.00059

**Published:** 2020-08-28

**Authors:** M. R. Chambers, R. Timothy Bentley, David K. Crossman, Jeremy B. Foote, Jey W. Koehler, James M. Markert, Nidal B. Omar, Simon R. Platt, D. Mitchell Self, Andy Shores, Donald C. Sorjonen, Alicia M. Waters, Amy B. Yanke, G. Yancey Gillespie

**Affiliations:** ^1^Department of Neurosurgery, University of Alabama at Birmingham, Birmingham, AL, United States; ^2^College of Veterinary Medicine, Purdue University, West Lafayette, IN, United States; ^3^Department of Genetics, University of Alabama at Birmingham, Birmingham, AL, United States; ^4^Department of Pathology, University of Alabama at Birmingham, Birmingham, AL, United States; ^5^College of Veterinary Medicine, Auburn University, Auburn, AL, United States; ^6^College of Veterinary Medicine, University of Georgia, Athens, GA, United States; ^7^School of Medicine, University of Alabama at Birmingham, Birmingham, AL, United States; ^8^College of Veterinary Medicine, Mississippi State University, Starkville, MS, United States; ^9^Division of Pediatric Surgery, University of Alabama at Birmingham, Birmingham, AL, United States

**Keywords:** oncolytic HSV (herpes simplex virus), checkpoint inhibition, Indoximod, IL-12, immunotherapy, canine (dog) glioma, one health, comparative oncology

## Abstract

As the most common and deadly of primary brain tumors, malignant gliomas have earned their place within one of the most multifaceted and heavily-funded realms of medical research. Numerous avenues of pre-clinical investigation continue to provide valuable insight, but modeling the complex evolution and behavior of these tumors within a host under simulated circumstances may pose challenges to extrapolation of data. Remarkably, certain breeds of pet dogs spontaneously and sporadically develop high grade gliomas that follow similar incidence, treatment, and outcome patterns as their human glioma counterparts. The most malignant of these tumors have been refractory to limited treatment options despite aggressive treatment; outcomes are dismal with median survivals of just over 1 year in humans and 2 months in dogs. Novel treatments are greatly needed and combination therapies appear to hold promise. This clinical protocol, a dose-escalating phase I study in dogs with sporadic malignant glioma, represents a first in comparative oncology and combination immunotherapy. The trial will evaluate M032, an Interleukin-12 expressing Herpes Simplex virus, alone and combined with a checkpoint inhibitor, Indoximod. Extensive pre-clinical work has demonstrated safety of intracranial M032 administration in mice and non-human primates. M032 is currently being tested in humans with high-grade malignant gliomas. Thus, in a novel fashion, both canine and human trials will proceed concurrently allowing a direct “head-to-head” comparison of safety and efficacy. We expect this viral oncolytic therapy to be as safe as it is in human patients and M032 to (a) infect and kill glioma cells, producing a virus and tumor cell antigen-rich debris field; (b) provide an adjuvant effect due to liberation of viral DNA, which is rich in unmethylated CpG sequences that “toggle” TLR-9 receptors; and (c) express IL-12 locally, stimulating induction of TH1 lymphocytes. The resultant immune-mediated anti-viral responses should, through cross-epitope spreading, translate into a strong response to tumor antigens. The ability to compare human and dog responses in real time affords the most stringent test of suitability of the dog as an informative model of human brain tumors. Subsequent studies will allow canine trials to properly inform the design of human trials.

## Introduction

### Malignant Gliomas

With an estimated incidence of 14.7 per 100,000 people in the United States and upward of 10,000–15,000 new annual cases, primary malignant tumors of the brain pose a formidable front in the world of oncology (1–3). Malignant gliomas are the most prevalent primary brain tumors, accounting for ~30% of all primary central nervous system (CNS) tumors in adults (4). In children, CNS tumors represent ~25% of all childhood malignancies and remain the leading cause of cancer-related morbidity and mortality (5). Interestingly, similar incidences of spontaneous central nervous system tumors have been observed in pet canines, at an estimated 14.5 per 100,000 animals (6). With a tendency toward certain breeds, up to 35% of these tumors that manifest represent gliomas that not only clinically behave like malignant gliomas in humans but also share similar radiographic, histopathologic, and genetic features with similar treatment and outcome patterns (7).

Current treatment paradigms for gliomas in both humans and pet dogs whose owners pursue therapy consists of a combination of maximum safe resection, chemotherapy, and/or radiotherapy. Tumors typically recur, result in disability, and ultimately lead to premature death.

### One Health

Advancement in field of brain tumor therapy has been woefully slow. Morbidity and mortality remain significant. A more correlative biologic approach is required for identifying pathogenesis and evaluating treatments.

Previous methods of studying gliomas in rodent models do not adequately represent features that define human cancers. For example, tumors in humans develop sporadically; have long periods of latency; complex biology of recurrence and metastasis, and varied responses to therapy. Murine models do not have comparable intra-tumoral heterogeneity and subjects often do not have an intact immune system. To overcome these deficiencies associated with the rodent model, many investigators have used spontaneous canine models to study human diseases.

Veterinary and human cancer patients have obvious clinical, anatomic, and histological similarities (8). Not only are tumor initiation and progression influenced by similar factors, but dogs also respond to and metabolize drugs in a manner comparable to humans (6, 9). Shared similarities between the two species include the incidence of sporadically occurring gliomas (6, 9–12), shared pathogenesis, and cancer-associated genetic alterations that influence cancer progression. Clinical symptoms of a brain tumor may include headache, hemiparesis, aphasia, sensory changes, memory loss, visual impairment, or seizures in humans and behavioral changes and seizures in dogs. Moreover, dogs have a natural 5 to 8-fold shorter lifespan than humans, resulting in a more rapid measurement of outcomes.

### Oncolytic Viral Therapy

**Figure 1:**
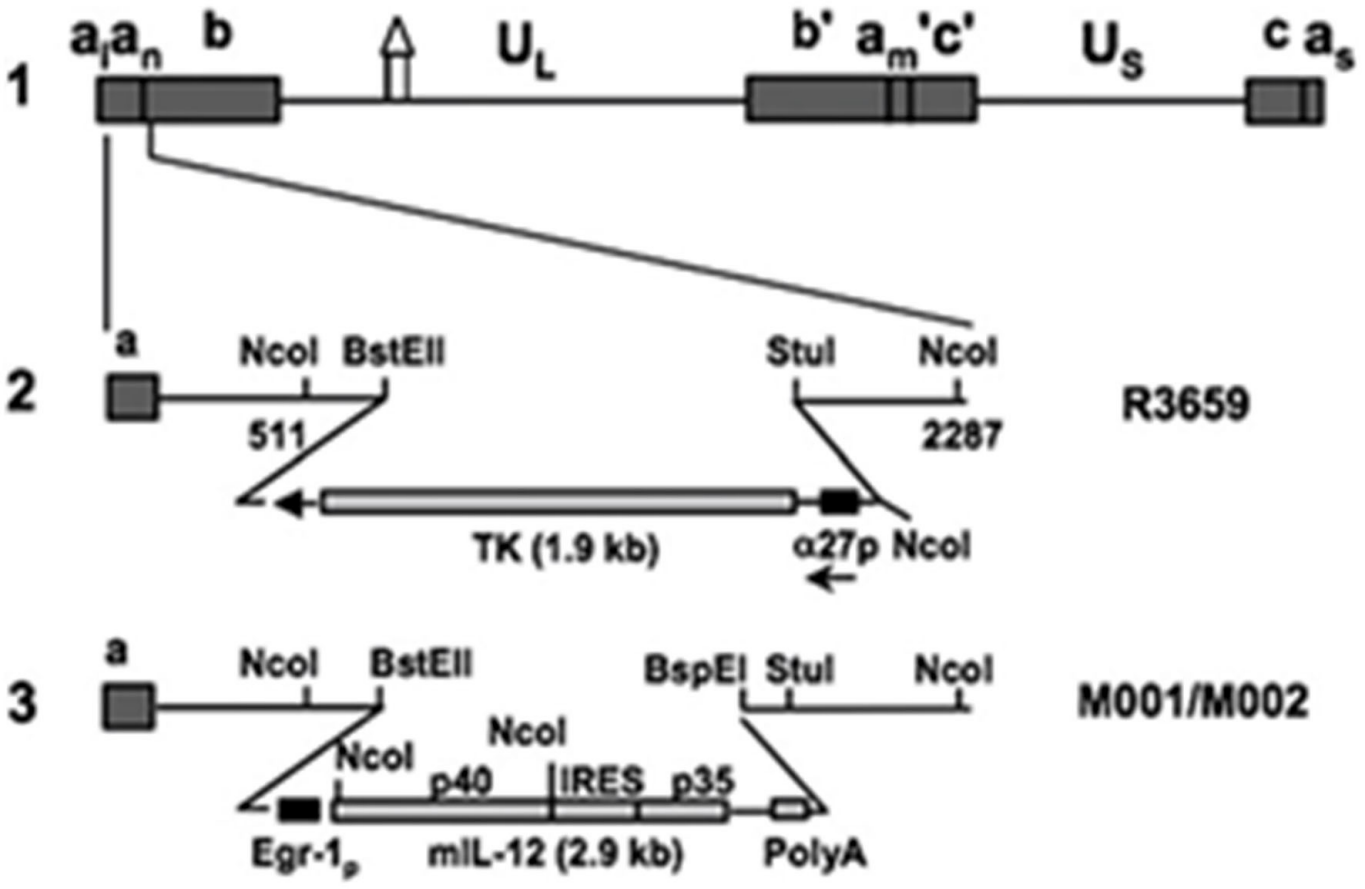
Murine interleukin 12-expressing HSV M002. Schematic representation of murine IL-12 expressing HSV (M002). Line 1 illustrates the HSV-1 (F) Δ305 genome, which contains a 700 bp deletion within the tk gene, as indicated by the symbol. U_L_ and U_s_ represent the unique long and unique short sequences, respectively. The inverted repeat sequences are indicated by *a, b*, and c, with subscripts n and m representing variable numbers of *a* sequences. a_1_ and a_s_ represent the α sequences flanking the U_L_ and U_s_ terminal repeats. Line 2 shows the sequence arrangement of the recombinant HSV R3659. The *BstEII-Stul* fragment within both copies of the γ_1_ 34.5 genes was replaced with the chimeric α27-tk gene in the inverted sequences ab (shown above) and b′a_m_ (not shown) flanking the U_L_ sequence. Line 3 shows the sequence arrangements of the relevant regions in the recombinant murine IL-12 expressing HSV M001 (tk–) or M002 (tk+). Ncol restriction sites are indicated. Copyright (2000) National Academy of Sciences, USA. Reproduced with permission form PNAS.

Over the past several decades, a number of oncolytic vectors have been studied in the pursuit of novel therapies for brain tumors. A few major subtypes include naked DNA, liposomes, and viruses; the latter of which include engineered forms of retrovirus, adenovirus (AdV), adeno-associated virus (AAV), and herpes simplex virus (HSV) type 1 (13). Targeted genetic recombination has allowed for tailored use of these viruses as oncolytic vectors in both pre-clinical (Figure 1) and clinical models (14).

HSV vectors possess the advantage of being innately neurotropic, and their candidacy as both safe and effective oncolytic agents has been established via numerous *in vitro* and *in vivo* studies of primary and metastatic brain tumors (15–19). The ability to harness genes necessary for neurovirulence and replication (20, 21) has greatly expanded the therapeutic potential of these viruses. Coding region alterations may render HSV vectors avirulent in post-mitotic brain tissue while allowing continued viral replication and killing of rapidly dividing tumor cells. With the γ_1_ 34.5 deletion, for example, HSV cannot overcome innate anti-viral response of normal cells and can only replicate in tumor cells. Selective replication in glioma cells makes HSV an invaluable weapon in the battle against malignant gliomas. This becomes particularly salient with the knowledge that upward of 10^9^ tumor cells may remain *in situ* following gross total surgical excision (13).

Like the murine Interleukin-12 homolog M002 (Figure 1), M032 is a similarly engineered novel oncolytic Herpes Simplex Virus (HSV) mutant expressing human Interleukin-12 (13, 22, 23) and infects and kills tumor cells, releasing IL-12 within the tumor bed to elicit a potent immune-related inflammatory response.

### Combination Immunotherapy

**Figure 2:**
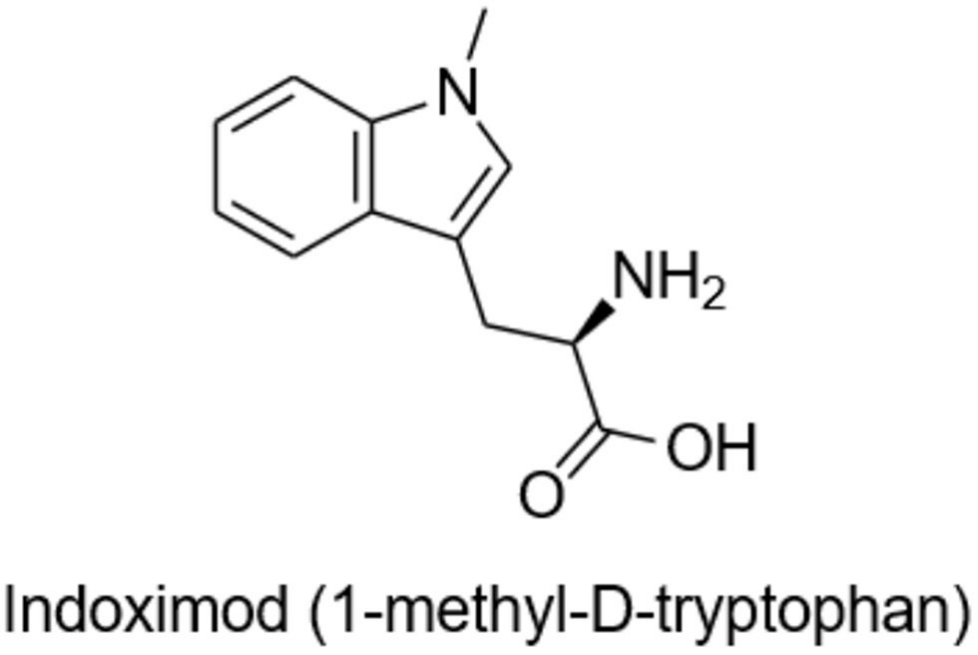
Indoximod (1-methyl-D-tryptophan).

M032 is highly antigenic, expresses IL-12, and unmethylated CpG sequences in its DNA are readily recognized by toll-like receptors (TLR) providing an adjuvant effect. Through cross-epitope spread, the initial anti-viral immune response is expected to evolve into an anti-tumor response. To amplify and prolong this beneficial response, we will test the ability of the small molecule immune checkpoint inhibitor, Indoximod (1-methyl-D-tryptophan), to down-regulate development of T regulatory cells (T-regs) and to promote the survival and continued activity of T effector cells (Figure 2).

Indoximod is an orally bioavailable checkpoint inhibitor that works systemically to inhibit indoleamine 2,3-dioxygenase (IDO), a key enzyme in the degradation of the essential amino acid tryptophan, resulting in immune checkpoint inhibitory activity. Tryptophan is important for differentiation of Treg lymphocytes, and its depletion can lead to immunosuppression via T cell arrest and anergy (24–26). Indoximod inhibits IDO at a Ki of 19 μM. TDO (tryptophan dioxygenase), IDO1, and IDO2 are inducible, rate-limiting enzymes that metabolize tryptophan to kynurenine, the effect of which is to immunosuppress CD8^+^ T effector cells, and to activate both CD4^+^ T-regs and myeloid derived suppressor cells (MDSCs). This is one of the previously described mechanisms by which high-grade gliomas evade immune surveillance (27, 28).

Several clinical trials investigating the use of Indoximod in adult cancer patients are underway [NCT01560923; NCT02073123; NCT02835729; NCT02052648 (completed); NCT03301636], as well as one in children with brain tumors (NCT02502708). Dose limiting toxicities have not been encountered at daily doses that completely inhibit the IDO1 enzyme. This rational approach includes administration of the IDO1 inhibitor during the initial 4 weeks of immune stimulation beginning with intratumoral virus administration, to allow a prolonged immune response. Our hypothesis is that Indoximod will blunt suppressive cellular immune response components (Tregs, MDSCs) and thereby allow a longer time for effective anti-viral and anti-tumor response that will be more durable than that observed with administration of the virus alone.

### Mechanism of Immune Tolerance in Response to IDO Inhibition

Tumor-induced host inflammatory responses can promote tumor secretion of indoleamine-pyrrole 2,3 dioxygenase (IDO). Expression of IDO in malignant gliomas correlates with increasing malignancy (29). Depletion of tryptophan leads to regulatory T-cell generation and activation, eliminating the tumor-targeting T cell response and allowing tumors to escape immune surveillance (26). The harnessing of this immunomodulatory pathway by these tumors may be overcome with IDO1 inhibitor-based treatments. Inhibition of IDO may act in both the tumor microenvironment and in draining lymph nodes and should result in production of more tumor-targeting T cells, re-establishing antitumor immunity. Several interventions inhibiting IDO1 have been shown to decrease tumor growth and elicit an immune response in rodent tumor models (30–34) but targeting IDO1 as a standalone treatment has often failed to cause tumor eradication and to prevent disease progression altogether. Likely, this failure occurs in antigenically “cold” tumors to which only a modest immune response has been mounted and inhibition of checkpoint mechanisms does little to amplify this effete response. It is more reasonable to use a highly antigenic virus to convert a tumor from “cold” to “hot” before expecting checkpoint inhibition to have the desired impact. There are several preclinical animal model studies of Indoximod in mice bearing syngeneic mouse gliomas that suggest an anti-tumor effect that is enhanced with chemotherapy (25, 35).

We are proposing to use Indoximod in the stage 2 component of this clinical immunotherapy trial in dogs since this isomer has been shown to be a competitive inhibitor of the IDO enzyme more specific for IDO1 than IDO2. Its toxicology and pharmacokinetics profile has been reported for dogs treated for 28 days by a daily oral dose of 600–1,200 mg/M^2^ (30–60 mg/kg) (24). Plasma half-life was 6 h with a T_max_ of 1 h and a bioavailability of 42%. There were no mortality events, adverse events, lesions identified on histopathology, or significant changes in body weight, hematologic or clinic chemistry values. For dogs, the dose of 1,200 mg/M^2^ was the NOAEL (No-Observed-Adverse-Effect-Level), but even doses above this level would not be expected to cause toxicity since the mean plasma concentrations would not increase. Thus, this is safe to administer to dogs with brain tumors and would not be expected to cause any toxicity with continuous daily dosing. Studies by Schmidt et al., suggest that, while the L-isomer of 1-Methyltryptophan (1-MT) is more effective in cell-free and *in vitro* studies, the D-isomer is superior in enhancement of anti-tumor immune responses *in vivo* (36). This paradoxical effect may be explained by a study by Opitz et al. (37) in which 1-D-MT increased IDO expression by ovarian cancer cell lines *in vitro*. Although investigators did not confirm this effect *in vivo*, this finding may be related to distinct gene expression profiles between long-term established cell lines and tumors *in situ*.

There are multiple active or completed human studies of Indoximod alone and in combination with chemotherapy or immunotherapy for advanced solid malignancies, including brain tumors (38–40). Newlink Genetics, Corp. has sponsored two companion clinical trials in children (NCT02502708) and adults (NCT02502648) with recurrent or progressive malignant brain tumors to explore safety and tolerability of Indoximod and temozolomide chemotherapy. Indoximod will be dose-escalated to define the Recommended Phase 2 Dose. The pediatric trial has been active and accruing since 2015 with an anticipated end date later in 2019. The adult trial reported (38) that 48 patients were treated by monotherapy with increasing doses (200–800 mg daily and 600–2,000 mg twice a day) and 5 patients demonstrated stable disease. The highest dose was 2,000 mg twice a day, but plasma levels plateaued at doses above 1,600 mg, similar to the pattern observed in the canine pharmacokinetic study. There were no dose limiting toxicities and only minor toxicities in the form of hypophysitis in three patients who had been previously treated with monoclonal antibody-based checkpoint inhibitors, further emphasizing Indoximod's safety profile.

### Preclinical and Clinical Safety and Efficacy Data

**Figure 3:**
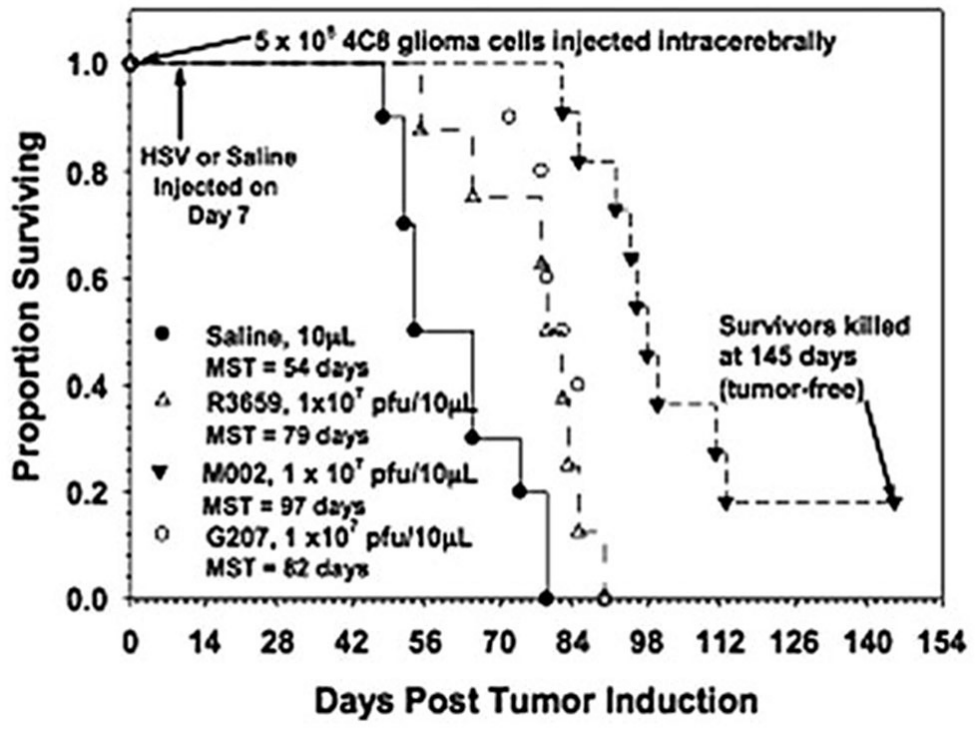
Survival in C57BL/6xDBA/2 Fhybrid mice injected with 4C8 mouse glioma cells followed by saline or HSV. Immunocompetent C57BL/6xDBA/2 F1 hybrid mice were injected intracranially with syngeneic 4C8 mouse glioma cells, followed in 7 days by saline or the viruses as shown. Median survivals were determined by Kaplan-Meier plots and are shown on the figure. Log rank analyses of these survival values confirmed the significantly prolonged survival-afforded mice treated with M002, compared with R3659 (*p* = 0.00007) or G207 (*p* = 0.0003). In addition, about 20% of the M002-treated mice were apparently “cured” with no evidence of remaining 4C8 glioma cells histologically when the experiment was terminated. Reproduced with permission from Oxford University Press.

**Figure 4:**
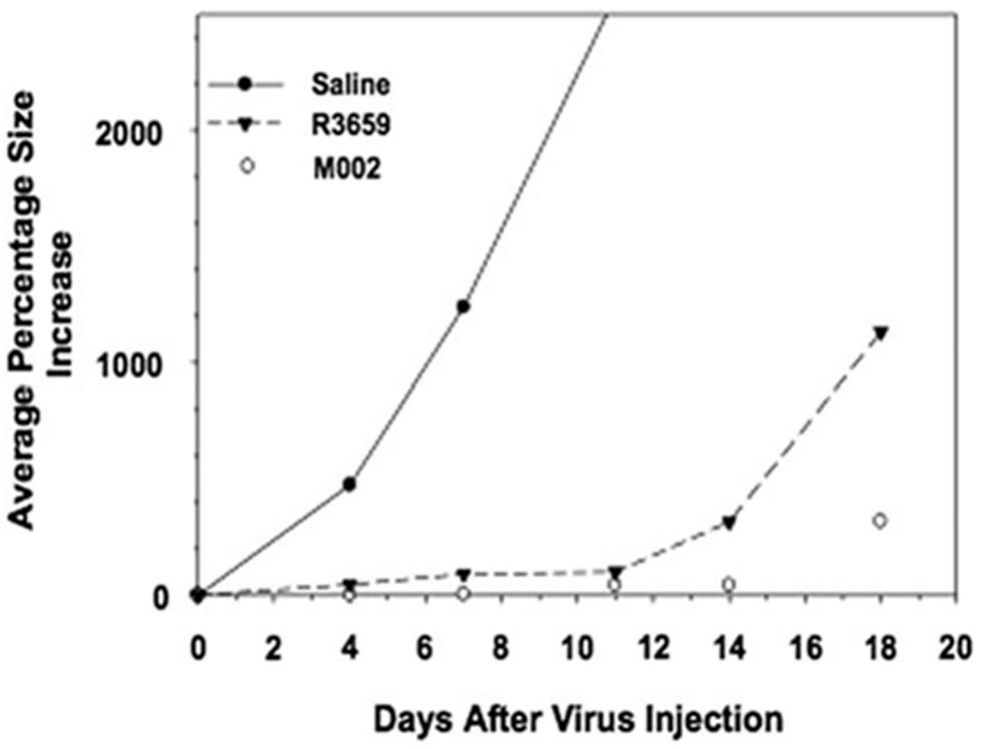
Tumor volumes after injection of Neuro-2A cells followed by saline or HSV in A/J mice. Immunocompetent A/J mice were injected subcutaneously with l × 10^5^ Neuro-2A cells. When tumors attained 200 mm^3^ in volume, they were injected with 50 μl of saline or 5 × 10^7^ pfu of HSV R3659 or M002. Tumor volumes were measured every 3 days, and the average percentage increase in volume was calculated relative to the treatment date. Reproduced with author permission from Patel et al. (13).

M002, an M032 variant expressing murine IL-12 (Figure 1), has been evaluated *in vivo* with several animal models (14, 22, 41). Mouse IL-12 (mIL-12) expression occurs at physiologically significant concentrations in tumors of neuroectodermal origin (from 800 to 3,200 pg/ml per 24 h/5 × 10^5^ cells) (13). In preclinical studies, M002 inoculation resulted in improved survival in immunocompetent syngeneic murine intracranial tumor models (Figure 3) and superior tumor growth inhibition in subcutaneous models (Figure 4) in comparison with both G207 (another clinically evaluated HSV) and R3659, the parental virus expressing no foreign genes (19).

Additional preclinical studies have further demonstrated increased efficacy of the murine IL-12-expressing oncolytic HSV (oHSV) M002 over the parent γ_1_ 34.5-deleted virus G207 (*not* expressing IL-12) in immunocompetent syngeneic intracranial mouse models of neuroblastoma and nude mice bearing human U87 malignant glioma xenografts (41). Immunohistochemical data have demonstrated augmented inflammatory responses in the immunocompetent syngeneic models exposed to the IL-12-expressing oHSV, suggesting that induction of a systemic immune response may at least partially account for anti-tumor responses (41).

Human IL-12 has been shown to be safe and effective in eliciting an immune response in animals, including dogs (42). While M002 expresses murine IL-12 and has not been previously utilized in canine clinical trials, its safety has been confirmed via *in vivo* testing in owl monkeys (*Aotus nancymaae)*. This species was chosen as they are highly sensitive to HSV, often terminally so. mIL-12 is also physiologically active in Aotus lymphocytes, further confirming their suitability as models for preclinical safety testing (22). Three *A. nancymaae* underwent intracerebral inoculation of purified M002 (produced using current GMP specifications), at titers of up to 4.8 × 10^8^ pfu, with no observed adverse effects. One treated female, along with a cage mate, demonstrated positive tuberculin skin tests almost five and a half years later that resulted in veterinarian-requested sacrifice of both animals. Necropsy, however, did not reveal evidence of tuberculous infection or viral toxicity in either animal. Another Aotus non-human primate (NHP) received a lower dose of non-purified M002 (standard laboratory preparation of 1 × 10^8^ pfu). This animal unexpectedly died while anesthetized for routine follow-up imaging. Necropsy demonstrated only age-related glomerulonephritis and bronchopneumonia that likely contributed to the untoward anesthetic reaction. Importantly, no signs of encephalitis or HSV-related toxicity were identified (23). One Aotus remained healthy for over 10 years following M002 administration. Routine MRI surveillance of the M002-treated monkeys never revealed any CNS abnormalities.

Biodistribution and toxicology of the human IL-12-expressing M032 virus were also determined *in vivo* by intracerebral injection of M032 HSV into 30 *A. nancymaae* NHPs, distributed evenly by sex, with an additional 6 NHPs serving as controls (saline injection). The virus was tested at both a low dose of 1 × 10^6^ pfu (human equivalent dose of 5 × 10^7^ pfu as determined via dose/gram of brain) and a high dose of 1 × 10^8^ pfu (human equivalent dose of 5 × 10^9^ pfu). Two male and two female NHPs from each dosing group were humanely euthanized at 3, 31, and 91 days. The animals underwent comprehensive necropsies as well as HSV PCR analyses to assess for the presence and copy numbers of the virus in various neural and systemic tissues. No serious toxicities or adverse events were identified that could be directly ascribed to M032 (23). Even in the single animal that required euthanasia 16 days after inoculation due to rapid deterioration of clinical condition, in which there was a question as to whether the test article was an inciting factor, no histopathologic evidence of herpes encephalitis was found and levels of the virus in the brain and systemic tissues were comparable to those of the other NHPs.

In summary, the M032 oHSV that will be utilized in this canine clinical trial is the product of rigorous molecular engineering and has undergone stringent preclinical safety testing for intracerebral inoculation, having been found to be safe in NHPs at per-kilogram doses well-exceeding those that will be used in this study.

As mentioned previously, the murine/canine study published by Jia et al. demonstrated no significant adverse effects with administration of Indoximod in dogs within a 28-day time period. Dose-escalation yielded an isolated mild to moderate increase in alkaline phosphatase levels (ALP) in dogs without concurrent changes in aspartate aminotransferase (AST) or alanine aminotransferase (ALT) levels (24). Rats exhibited what were described as significant but sporadic increases in globulin levels, decreased ALT levels, decreased creatinine levels, and increased blood urea nitrogen (BUN), glucose, total protein, and cholesterol levels that canine patients did not exhibit. Mild variations in hemoglobin and red blood cell levels in dogs and eosinophil and lymphocyte levels in rats were not felt to be treatment-related. Clinically, the authors mention “sporadic gastrointestinal events” in dogs that were not dose-dependent, and nasal discharge in rats that was not considered significant. Necropsy revealed small non-specific pulmonary, renal, splenic, and cutaneous lesions with multi-organ microscopic lesions that were also sporadic and not believed to be related to treatment. The authors point out that the eosinophilia-myalgia type syndrome previously described in humans as well as Lewis rats receiving L-tryptophan derivatives was not observed in Lewis rats or canines receiving Indoximod, a D-tryptophan derivative.

#### Study Objectives

The primary objectives of this study are to ascertain the safety and tolerability of a single intracranial injection of the M032 virus (NSC 733972) in pet dogs with spontaneously occurring high grade gliomas following maximum safe surgical resection, at escalating doses in consecutive cohorts of canines, in order to delineate the maximum tolerated dose (MTD) of M032 and to further establish safety and tolerability of the viral therapy in combination with the checkpoint inhibitor Indoximod.

Secondary objectives include (1) obtaining provisional data regarding potential progression-free and overall survival benefit of M032 in the treatment of canine patients with high grade gliomas, both alone and with added IDO inhibition; (2) establishing baseline (pre-treatment) local and systemic immune profiles and characterizing immune responses referable to M032 administration without and with IDO inhibition; and (3) measuring *in situ* activity of the M032 virus following inoculation. These secondary objectives are dependent on obtaining tumor tissues from clinically indicated re-operation and/or necropsy.

#### Study Design

This two-stage Phase 1 open-label dose-escalating clinical trial will evaluate responses to a clinical-grade preparation of M032 (NSC733972), an oncolytic, IL-12-expressing, HSV (13, 22, 23) alone in Stage 1 and in combination with Indoximod (1-methyl-D-tryptophan) in Stage 2.

#### Subject Selection and Withdrawal

##### Inclusion criteria

Study subjects must meet the following inclusion criteria to be included (Table 1).

**Table 1:** Inclusion criteria.

1. Pathologically proven malignant glioma 2. Canine subjects must have clinical and MR imaging findings consistent with a diagnosis of malignant glial tumor. 3. Age >6 months. 4. Modified Karnofsky Performance Status (MKPS) >50 (see Appendix A in Supplementary Material). 5. Life expectancy of >6 months. 6. Ability of pet owners to understand and the willingness to sign a written informed consent document. 7. Steroid use is allowed as long as the dose has not increased within 2 weeks of scheduled M032 administration and the dose is equivalent to a dexamethasone equivalent dose of ≤ 2 mg/kg/day at the time of treatment.

##### Exclusion criteria

Subjects that meet the following criteria are excluded (Table 2).

**Table 2:** Exclusion criteria.

1. Bitches must not be pregnant; this will be confirmed by a negative serum pregnancy test within 14 days prior to starting study treatment. Pregnant or nursing bitches are excluded from this study because M032 is a viral oncolytic therapy with unknown potential for teratogenic or abortifacient effects. There is an unknown but potential risk for adverse events in nursing pups secondary to treatment of the bitch with M032. 2. Subjects who have had chemotherapy, cytotoxic therapy, immunotherapy within 4 weeks prior to entering the study (6 weeks for nitrosoureas), surgical resection within 4 weeks prior to entering the study, or have received experimental viral therapy or gene therapy at any time (e.g., adenovirus, retrovirus, or herpes virus protocol) will be excluded. However, this does not preclude re-treatment with M032 at a later date. 3. Subjects who have not recovered from adverse events due to therapeutic interventions administered more than 4 weeks earlier will be excluded until they have recovered. 4. Subjects may not be receiving any other investigational agents. 5. History of allergic reactions attributed to compounds of similar biologic composition to M032 or to IL-12. 6. Tumor involvement that would require ventricular, brainstem, basal ganglia, or posterior fossa inoculation or would require access through a ventricle in order to deliver treatment. 7. History of encephalitis or other CNS infection. 8. Required steroid increase within 2 weeks of scheduled M032 administration. When possible, the subject should be on a dexamethasone equivalent dose of ≤ 2 mg/kg/day at the time of treatment. 9. Active herpes lesion or concurrent therapy with any drug active against HSV (acyclovir, valacyclovir, penciclovir, famciclovir, ganciclovir, foscarnet, cidofovir). 10. Uncontrolled intercurrent illness including, but not limited to ongoing or active infection, symptomatic congestive heart failure, unstable angina pectoris, cardiac arrhythmia, or any other medical condition that precludes surgery. 11. History of allergic reaction to intravenous contrast material not amenable to pre-treatment. 12. Subjects with pacemakers, ferromagnetic implants, metal infusion pumps, metal pellets, or shrapnel fragments or certain types of stents.

##### Subject recruitment and screening

The Phase I clinical trial will be conducted at multiple collaborating U.S. Colleges of Veterinary Medicine. Veterinarians and study personnel at each veterinary center will perform recruitment and enrollment upon identification of a potential study subject. Presumptive diagnosis of glioma is made by history, physical and neurologic examinations, and brain MR imaging. Screening will occur within 2 weeks of treatment initiation. At the time of screening, informed consent will be obtained from the owner(s), inclusion and exclusion criteria will be reviewed to confirm eligibility for enrollment, and comprehensive veterinary evaluation including protocol-specific imaging and laboratory testing will be conducted. The study is expected to enroll ~12 subjects per year.

##### Informed consent

The general principles underlying the therapeutics strategy of this trial will be explained in non-medical, lay language to the pet dog subject owners. Potential risks, benefits, indications for and alternatives to enrollment, as well as expected time commitments will be discussed. The dog owner(s) will be provided with a summary of this information and those who decide not to enroll in this trial will be offered conventional standard of care treatment(s). Appendix C in Supplementary Material includes one example of a consent form that will be utilized during enrollment in this study.

## Materials and Equipment

### Study Drugs

#### M032 Dose and Administration

In Stage 1, dosing of M032 will begin at a level of 1 × 10^5^ plaque forming units (pfu), and the dose will be escalated in subsequent tri-canine cohorts by one-log increments until either the dose of 1 × 10^9^ pfu is reached or an unacceptable incidence of dose-limiting-toxicities (DLT) is encountered (Table 3). Any dosing cohort in which a DLT is encountered will be expanded to include six subjects. Two DLTs occurring at any given dose level will automatically define the MTD. Otherwise, dose escalation will proceed per the outlined criteria. If no MTD is defined by this design, the maximally planned dose will be considered to be the Recommended Phase II Dose (RP2D) for subsequent studies.

**Table 3:** Dose escalation decision criteria.

**Number of patients with dose-limiting toxicity (DLT) at given dose level**	**Escalation decision rule**
0 out of 3	Enter 3 patients at the next dose level
>/= 2	Dose escalation will be stopped. This dose level will be declared the maximally tolerated dose (highest dose administered). Three additional patients will be entered at the next lowest dose level if only 3 patients were treated previously at that dose
1 out of 3	Enter up to 3 more patients at this dose level -If 0 out of these 3 patients experience DLT, proceed to the next dose level -If 1 or more of this group suffer DLT, then dose escalation is stopped, and this dose is declared the maximally administered dose. Three additional patients will be entered at the next lowest dose level if only 3 patients were treated previously at that dose
< /= 1 out of 6 at highest dose level below the maximally administered dose	This is generally the recommended phase 2 dose. At least 6 patients must be entered at the recommended phase 2 dose

In the second stage of this protocol, we will reduce the initial dose by one log from the MTD/RP2D level to compensate for any unknown effects that might be associated with an enhanced immune response due to the checkpoint inhibitor (Table 4).

**Table 4:** Dose-escalation schedule for canine patients.

**Dose level**	Dose of MO32^*^ **plaque forming units (pfu)**	
	**Stage 1**	**Stage 2**
Level −1	1 × 10^4^ pfu	
Level 1	1 × 10^5^ pfu	
Level 2	1 × 10^6^ pfu	
Level 3	1 × 10^7^ pfu	
Level 4	1 × 10^8^ pfu	1 × 10^8^ pfu + 1,200 mg/M^2^ Indoximod
Level 5	1 × 10^9^ pfu	

* *Doses are stated as exact doses in plaque forming units rather than as a percentage*.

#### Indoximod Dose and Administration

During stage two, the dogs will receive 1,200 mg/M^2^ (60 mg/kg) of Indoximod orally as a single starting dose, followed by 600 mg/M^2^ (30 mg/kg) twice daily for a period of 28 days.

## Methods

### Study Procedures

All subjects will be prescribed anti-convulsant medication following screening, prior to initiation of subsequent study procedures. This anti-convulsant (levetiracetam, 500 mg daily) will be continued for a minimum of 6 weeks unless the subject exhibits an adverse reaction (43). Thereafter, the anticonvulsant may be weaned or discontinued at the discretion of the attending veterinarian.

After screening, preoperative assessment and informed consent as described above, all subjects will receive general anesthesia and undergo a gross total or partial surgical resection of tumor by veterinary neurosurgeons at Day 0. A catheter will be placed intraoperatively, with the distal end positioned in the tumor bed and the proximal end secured to a subcutaneous reservoir to be used for infusion. Post-operative CT imaging will be performed to confirm appropriate catheter placement. If a catheter is determined to be out of position by the lead investigator, then the catheter will be repositioned, with placement again confirmed by CT imaging. The subcutaneous reservoirs and catheters will be left in place for up to 3 months to serve as imaging fiducials or indefinitely (after 3 months, they may be removed if necessary. Placement is subcutaneous and removal may be performed in a clinic setting as an outpatient surgery with sedation).

Each dog will be continuously monitored after surgery in the veterinary intensive care unit until they have regained consciousness and maintain normal temperature, heart rate, respiratory rate, and blood pressure. They will then be monitored per protocol at each veterinary site until discharge, typically on post-operative day 3 or 4. Distress of pain is not anticipated during any of these phases but should there be any pain behaviors, the subjects will be immediately treated with partial opioids (butorphanol or buprenorphine), opioids (fentanyl), alpha-agonists (dexmedetomidine), non-steroidal anti-inflammatory medications (carprofen), and/or tramadol unless contraindicated.

#### Stage I

Within 48 h of surgery, intratumoral infusion of M032 will begin. Provided no unacceptable toxicities are encountered, at least one cohort of three animals will be enrolled at each dosing level. Dose escalation decision criteria are outlined in Table 3 and the dose escalation schedule is described in Table 4. Based on each subject's dosing cohort, the indicated dose of M032 will be administered in a standardized volume of ~0.5 ml through the catheter via percutaneous injection into the subcutaneous reservoir, followed by a 0.5 ml infusion of sterile vehicle (Dulbecco's Phosphate-Buffered Saline plus 10% glycerol; Avanti Pharmaceutical).

#### Stage II

Once the MTD/RP2D of M032 has been established, subsequent dogs will receive M032 of 1 log below this dose and 4 weeks of daily Indoximod following surgery, catheter placement and CT confirmation.

### Surveillance and Immunologic Monitoring

An in-depth analysis of immune response is needed to understand the effects of M032 alone and combined with checkpoint inhibition. The study schedule is shown in Table 5. Following surgical resection of tumor and intratumoral infusion of M032, animals will be assessed at regular intervals (days 1, 2, 3, 14, and months 1, 3, 6, 9, and 12) with quality of life assessments, physical and neurologic examinations, and laboratory studies to monitor objective response to ongoing treatment. Preoperative screening MRI will serve as the baseline imaging study and follow-up scans will be performed at study day 28, and at months 3 and 6. Intratumoral changes will also be assessed indirectly by evaluating plasma levels of cytokines and shifts in immune related circulating lymphoid cells. Blood, saliva, and conjunctival smears will be collected. HSV detection will be performed. These studies will provide valuable information about the immune response of the dog to the HSV immunotherapy and will be extraordinarily useful in Stage II with the addition of the checkpoint inhibitor. After discharge, all subjects will be followed closely for evidence of adverse events. In addition to monitoring for signs of any adverse events, subjects in Stage 2 will have blood samples drawn to measure serum/plasma baseline and post-Indoximod kynurenine levels longitudinally by ELISA to determine any correlations with immune profile changes. Data from stage I will be used to determine the extent to which the dog's immune system is responding differently to the combination therapy.

**Table 5:** Study schedule.

	**Pre-study (within 2 weeks prior to MO32 administration)**	**Day 0**	**Day 1**	**Day 2**	**Day 3**	**Day 14 (+/– 4 days)**	**Day 28 (+/– 4 days)**	**Month 3 (+/– 12 days)**	**Month 6 (+/2 12 days)**	**Month 9 (+/– 12 days)**	**Month 12 (+/– 12 days)**
Informed consent	X										
Inclusion/exclusion criteria	X										
Signalment	X										
Pregnancy test (serum)	X										
Medical history	X										
Concurrent meds	X	X	X	X	X	X	X	X	X	X	X
Complete physical exam (with wound assessment Days 1–14)	X		X	X	X	X	X	X	X	X	X
Vital signs	X	X	X	X	X	X	X	X	X	X	X
CBC, c diff, platelets	X			X		X	X	X	X	X	X
Serum chemistries, PT/PTT, INR	X			X		X	X	X	X	X	X
Kynurenine levels (serum)	X			X		X	X	X	X	X	X
EKG	X										
CXR (AP and lateral)	X										
Urinalysis with micro	X										
Adverse event evaluation		X	X	X	X	X	X	X	X	X	X
MRI	X						X	X	X		
Neurologic exam^**^	X	X	X	X	X	X	X	X	X	X	X
HSV Ab titer	X					X	X	X	X	X	X
HSV detection (saliva, conjunctival secretions, and/or blood)	X			X	X^*^	X	X	X	X	X	X
IL-12 detection (serum)	X			X	X	X	X	X	X	X	X
MO32 administration		X									
Blood for LTA, Elispot	X			X	X	X	X	X	X	X	X
Blood to be stored for future immune studies	X	X		X	X	X	X	X	X	X	X
Head CT		X									
Surgery, catheter placement		X									
Levetiracetam (prophylactic AED) daily ×6 weeks beginning prior to Day 0^***^	X										
Indoximod administration, daily ×28 days beginning at Day 0^****^		X									

* *Blood only*.

** *Appendix B in Supplementary Material: Modified Glasgow Coma Scale (MGCS)*.

*** *AED discontinued prior to 6 weeks if adverse reaction. After 6 weeks, continuation is at the discretion of attending veterinarian*.

**** *Stage 2 of trial*.

Proliferation of T (CD3^+^ CD4^+^ and CD3^+^ CD8^+^) and NK (CD3^−^ CD94^+^ Nkp46^+^) cells within isolated peripheral blood mononuclear cells (PBMCs) will be evaluated and compared pre and post-treatment. Specifically, PBMC (2 × 10^6^) will be labeled with CFSE (carboxyfluorescein succinimidyl ester) and then cultured for 72 h alone or in the presence of UV-inactivated M032 (1 × 10^8^ pfu/ml). Cells will be analyzed by flow cytometry for proliferation by CFSE dilution. Second, PBMCs will be cultured for 72 h alone or in the presence of M032 and assayed by flow cytometry and ELISpot for expression of IFN-γ (T_H_1) and IL-4 (T_H_2). Distribution of immune cell types within peripheral blood will be assessed using multi-parameter flow cytometry with a focus on T (CD3+ CD4^+^ Foxp3^−^ T helper, CD3+ CD4+ Foxp3+ regulatory, and CD3^+^ CD8^+^ cytotoxic T cells), B cells (CD79a^+^ CD21^+^ IgG^+/−^), NK cells (CD94^+^ NKp46^+^), neutrophils (CD11b^+^ CAD048A^+^ MHCII^low^ CD14^−^), and monocytes (CD11b^+^, CD14^+^, MHCII^low/int^). The activation status of T and B cell subsets will be assessed using antibody combinations to elucidate naïve (CD44^lo^ CD62L^hi^), effector (CD44^hi^ CD45RA^lo^ CD62L^lo^), and central memory (CD44^hi^ CD45RA^lo^ CD62L^hi^). We will also quantify total serum IgG and HSV-specific IgG antibody titers and determine whether HSV-specific antibodies can induce NK cell-mediated antibody-dependent cellular cytotoxicity (ADCC) as an indicator of antitumor immunity using *in vitro* cell based assays. Antibodies to HSV from the serum will be co-incubated with purified NK cells and tumor cells at various NK:tumor cell ratios to determine whether HSV-specific antibodies can modulate NK cell-mediated tumor cell lysis.

Systemic immune distribution of HSV will be evaluated through direct assessment of serum HSV-titers, *in vitro* culture, quantification of human IL-12, and quantitative PCR for HSV dsDNA. The presence of HSV in saliva and conjunctival samples will be evaluated by qPCR.

### Tumor Analysis

High quality, appropriately processed tumor tissue from all subjects will undergo histologic analysis by three independent veterinary pathologists. Routine histopathology will be compared to extensively described histopathologic features of human tumors and based on the classification scheme proposed by Koehler at al. (44). We will conduct immunohistochemistry of recurrent tumor resected prior to HSV infusion and on any tumor tissue obtained from subsequent surgical resection or necropsy. We are interested in infiltration of various subsets of immune-related inflammatory cells (as described above) as well as changes in expression of proliferation markers (Ki-67), endothelial cell markers (vWF, CD31) and the presence of any HSV virus antigens.

## Results

### Statistical Plan

Descriptive statistics will be used to summarize the safety and efficacy variables collected, the baseline demographic data, and any adverse events with careful analysis of any effects that M032 may have on progression-free and overall survival, neurologic function, and behavior, or the development of herpes infection. The occurrence and frequency of all adverse events will be determined. Specifically, any event meeting the criteria for a grade 3 toxicity or greater and any event related to the study drugs will be carefully denoted.

Categorical variables will be represented as absolute frequency counts and percentages. For continuous variables, including applicable demographic and baseline characteristics for each cohort, standard descriptive statistical measures including mean, median, standard deviation, and range will be employed. Values for discrete factors will be tabulated as is.

Normative canine data and baseline pre-treatment values from each dosing cohort will serve as references for analysis of laboratory data, and group means/standard errors will be determined. When indicated, logarithmic transformations will be applied. Univariate and multivariate analyses will be conducted for any concurrent medications, illnesses, or prior cancer treatments to identify potential confounders in the treatment-response relationship.

Additional analyses of the data on secondary endpoints, including measurements of mononuclear cells, serum cytokines, and other serum/plasma defined inflammatory/infection indicators will delineate local and systemic immune responses to the M032 virus, progression-free survival, overall survival alone and with IDO inhibition. Routine bioinformatic analyses of exome sequencing and transcriptomic (RNA-Seq) sequencing data from tumor samples will be performed.

Both mRNA and exome sequencing will be performed on the Illumina NextSeq500, as outlined by the manufacturer (Illumina Inc., San Diego, CA). Exome capture will be done using the Agilent SureSelect Canine capture kit (Agilent). For exome analysis, the raw sequence files will be analyzed using Broad's Genome Analysis ToolKit (GATK) following their Best Practices Guideline, which will return variants of interest in these tumors. For mRNA-Seq, STAR will be used to align the raw sequences to the canine genome followed by HTSeq-Count to estimate transcript abundances, followed by DESeq2 to normalize and calculate differentially expressed genes to identify genes up-/down-regulated in the tumors.

### Safety and Adverse Events Analyzed

Following discharge from the veterinary facility, the subject will undergo close outpatient follow-up and monitoring for adverse events, as shown in the study schedule, with additional evaluations as medically indicated. Any serious, life-threatening, suspected or unexpected adverse event that occurs during the conduct of this study, regardless of relationship to test article or procedures, will be reported in writing to the lead investigators, the Institutional Animal Care and Use Committees, and the Institutional Biosafety Committees.

Euthanasia may represent a humane endpoint used to eliminate pain and/or distress. As set forth in the “AVMA Guidelines for the Euthanasia of Animals: 2013 Edition, Members of the Panel on Euthanasia: 13.1 A Good Death as a Matter of Humane Disposition,” three components are used to describe animal welfare: that the animal “(1) functions well, (2) feels well, and (3) has the capacity to perform behaviors that are innate or species-specific adaptations” (45). These three components will be used to assess each animal at each time-point. In addition, medical and physical examination measures, including but not limited to vital signs, continence, cognition, appetite, presence of medically uncontrollable seizures, evidence of intractable discomfort, pain, or suffering will all be considered and weighed together with the experience and judgment of the attending veterinarian and observations of the pet owners.

## Discussion

### Risk–Benefit Assessment

The study's primary objective is to determine the safety and tolerability of intratumoral injection of M032 alone (Stage I) and in concert with the checkpoint inhibitor Indoximod (Stage II) in canines with malignant gliomas. Although M032 has not been associated with adverse events in humans, given its limited use to date it is plausible that it might result in adverse events comparable to those observed in phase I trials of G207, a similarly genetically engineered HSV. Documented adverse effects related to administration of G207 included: headache, nausea, drowsiness, weakness, anemia, and leukopenia [Appendix D in Supplementary Material includes a list of adverse events and related international medical terminology (IMT) terms]. If there is concern for encephalitis, a CT scan or MRI scan will be performed and the antivirus drug acyclovir administered if the diagnosis is supported. Indoximod was not shown to have specific adverse clinical effects in dogs in the preclinical study by Jia et al. but these results are limited by the short time period of observation (28 days), histopathologic studies being performed on only one male and one female at each dose, all eight canines being purpose-bred beagles, and the lack of description of histopathological analysis of synovium or other joint tissues. The more commonly described adverse events in humans receiving Indoximod include fatigue, anemia, anorexia, dyspnea, cough, and nausea. Appendix E in Supplementary Material includes a list of possible adverse events associated with Indoximod use and related IMT terms, based upon those reported to occur in ≥10% of cases in the Phase I human study by Soliman et al. (38). There may also be unpredicted side effects to treatments in this study. Participation in this study may not be of any direct medical benefit to the canine subjects. If a subject responds to this therapy, the tumor may shrink and the symptoms caused by the tumor may improve. We expect that there may be an improvement in the canine's quality and quantity of life. Furthermore, there may be indirect benefits; information learned from this study may help both canines and humans with brain tumors in the future.

## Conclusion

From within the robust, multifaceted field of brain tumor research, we present a premier example of the exciting comparative oncology approach in our work with the naturally occurring canine glioma model. Capitalizing on the pathophysiologic and genetic similarities between glial tumors in humans and dogs, our consortium has designed the first Phase 1 clinical trial tailored to canine patients using the same oncolytic herpes virus concurrently being utilized in a human clinical trial for recurrent high grade gliomas in adults. Rooted in high quality pre-clinical safety and efficacy data, this trial will delineate the safety and tolerability of oncolytic virus administration in dogs that harbor spontaneously-occurring gliomas and will also provide insight into the efficacy of the novel application of this treatment with and without the adjunctive use of an immune checkpoint inhibitor. Through state of the art exome sequencing, histologic analysis, and immunologic monitoring, we will reveal how these tumors and the immune system are impacted by the therapy, synthesize the data and compare with those acquired from human trials to inform the design of future prospective, randomized control trials. The overarching goal of our work is to not only improve survival and quality of life in pet dogs with glial tumors, but also to inform human studies and improve outcomes in both species in bi-translational fashion. Thus, we undertake this rigorous and innovative comparative oncology effort to evaluate combination immunotherapy in the treatment of this devastating disease.

## Ethics Statement

The UAB coordinating enter has obtained IACUC approval to oversee and supervise this study in companion dogs with brain tumors and each participating College of Veterinary Medicine has obtained approval to conduct the study from their respective Institutional Animal Care and Use Committee (IACUC).

## Author Contributions

MC, RB, DC, JF, JK, JM, SP, AS, DCS, AY, and GG contributed the conception and design of the study. MC wrote the first draft of the manuscript. NO, DMS, and AW wrote sections of the manuscript. All authors contributed to manuscript revision, read, and approved the submitted version.

## Conflict of Interest

JM and GG are founders of and own stock and stock options (<8% interest) in Aettis, Inc., a biotech company that is developing other oHSVs that are not the subject of this current investigation. GG currently serves as one of five unpaid members of the Board of Directors for Aettis, Inc. JM and GG are a founders of and own stock and stock options (<25%) in TreoVir, LLC, which has licensed G207 HSV that is not the subject of the current investigation. JM and GG were also founders of and owned stock and stock options (<8%) in Catherex Inc., a biotechnology company that had licensed additional intellectual property related to oHSV. Catherex, Inc., was sold to Amgen, Inc., on December 18, 2015, and they no longer participate in any decision making or have any control of any aspect of Catherex or Amgen, although they have received proceeds from the sale of the company in a structured buyout. GG has served as a paid advisor to the Program Project at the Ohio State University that seeks to find improved methods for application of distinct oHSV to treat localized and metastatic cancers. This is generally, but not specifically, related to the subject matter of this investigation. JM also holds intellectual property in another oHSV not the subject of this current investigation that has been licensed by Mustang Biotech, Inc. The remaining authors declare that the research was conducted in the absence of any commercial or financial relationships that could be construed as a potential conflict of interest.

## Abbreviations

ALP: alkaline phosphatase
ALT: alanine aminotransferase
AST: aspartate aminotransferase
BUN: blood urea nitrogen
CNS: Central Nervous System
GBM: glioblastoma multiforme
CpG: cytosine triphosphate deoxynucleotide phosphodiester-linked to guanine triphosphate deoxynucleotide; recognized by Toll Receptor 9
HSV: herpes simplex virus
oHSV: oncolytic HSV
M002: mutant γ1 34.5-deleted oHSV engineered to express mouse Interleukin-12
M032: mutant γ1 34.5-deleted oHSV engineered to express human Interleukin-12
G207: doubly mutant oHSV; both copies of γ1 34.5 gene have been deleted and virus ribonucleotide reductase (UL39) has been insertionally inactivated
IDO: indoleamine 2,3-dioxygenase
AdV: adenovirus
AAV: adeno-associated virus
TDO: tryptophan dioxygenase
Tregs: regulatory T cells
MDSCs: myeloid derived suppressor cells
MTD: maximum tolerated dose [in North America, the maximum tolerated dose (MTD) is the RP2D; elsewhere, the MTD is considered the dose level above the RP2D]
RP2D: Recommended Phase 2 Dose (usually the highest dose with acceptable toxicity, defined as the dose level producing around 20% of dose-limiting toxicity)
SGOT: serum glutamic-oxaloacetic transaminase (AST)
NOAEL: no-observed-adverse-events-level
PCR: polymerase chain reaction
MKPS: modified Karnofsky performance status
DLT: dose-limiting toxicity
GMP: good manufacturing practice
CFSE: carboxyfluorescein succinimidyl ester
FACS: fluorescence-activated cell sorting flow cytometry
IACUC: Institutional Animal Care and Use Committee
IBS: Institutional Biosafety Committee
ADCC: antibody-dependent cellular cytotoxicity
NK cell: natural killer cell
CD3: cluster of differentiation antigen 3—expressed on mature T lymphocytes
CD4: cluster of differentiation antigen 4—expressed on helper T lymphocytes
CD8: cluster of differentiation antigen 8—expressed on cytotoxic T lymphocytes
CD56: cluster of differentiation antigen 56—expressed on activated CD8+ T and γδ T lymphocytes and dendritic cells
CD25: cluster of differentiation antigen 25—Interleukin 2 receptor alpha expressed on activated T and B lymphocytes, myeloid precursors and resting memory T cells
FoxP3: forkhead box protein 3 expressed on natural and adaptive/induced T regulatory cells
CD79a: cluster of differentiation antigen 79A—B cell receptor-associated alpha chain
CD21: cluster of differentiation antigen 21—B cell complement receptor type 2
CD11: cluster of differentiation antigen 11—integrin alpha component
CD44: cluster of differentiation antigen 44—cell-surface glycoprotein on many cell types involved in cell-cell interaction, migration, cell adhesion
CD45RA: cluster of differentiation antigen 45RA—protein tyrosine phosphatase receptor C, leukocyte common antigen
CD62L: cluster of differentiation antigen 62L—L-selectin, a cell adhesion molecule found on lymphocytes; important in lymphocyte-endothelial interactions.
