# Supplementary material for: The One Health Consortium: Design of a Phase I Clinical Trial to Evaluate M032, a Genetically Engineered HSV-1 Expressing IL-12, in Combination With a Checkpoint Inhibitor in Canine Patients With Sporadic High Grade Gliomas
Source: Front Surg. 2020 Aug 28;7:59. doi: 10.3389/fsurg.2020.00059 (PMC7484881; doi:10.3389/fsurg.2020.00059)
Supplement: Supplementary file 1 [file Table_1.pdf]

Appendix A – Performance status scale (Human Karnofsky Performance Score and Karnofsky score adapted for dogs)

**Human Karnofsky Performance score and Karnofsky score adapted for dogs**		
**Score**	**Karnofsky score in human medicine**	**Karnofsky score adapted for dogs-Canine Karnofsky score (CKS)**
**100**	Normal, no complaints, no evidence of disease	Normal activities of daily living (ADLs); no clinically evident alterations even with the presence of a small, asymptomatic tumor
**90**	Able to carry on normal activity; minor symptoms of disease	Normal ADLs; one organ with localized accompanying disease of minor importance
**80**	Normal activity with effort; some symptoms of disease	Normal ADLs; two organs with localized accompanying disease of minor importance
**70**	Cares for self; unable to carry on normal activity or active work	At least once organ with accompanying disease of major importance. Mild lethargy over baseline (Lethargy Grade 1), normal appetite
**60**	Requires occasional assistance but able to care for needs	At least one organ with accompanying disease of major importance. Moderate lethargy causing some difficulty with performing ADLs (Lethargy Grade 2). Slightly decreased appetite but normal water intake
**50**	Requires considerable assistance and frequent medical care	At least one organ with accompanying disease of major importance. Lethargy Grade 2. Moderately decreased appetite but normal water intake
**40**	Disabled; requires special care and assistance	Severely restricted in ADLs; only ambulatory to the point of performing ADLs (Lethargy Grade 3). Severely decreased appetite. Spontaneous water intake slightly reduced, but without clinical signs of dehydration
**30**	Severely disabled; hospitalization, death not imminent	Lethargy Grade 3. Animal still stands up spontaneously and remains standing for a while; restraining necessary during examinations or therapy. Severely decreased appetite and spontaneous water intake, clinical signs of dehydration
**20**	Very sick; hospitalization necessary, active treatment necessary	Disabled; must be force-fed and helped to perform ADLs (Lethargy Grade 4). Remaining most of the time in lateral decubitus and stands up only for defecation and urination. Slight restraining for examination or treatment still necessary
**10**	Moribund; fatal process progressing rapidly	Absent ADLs; dog remains constantly in lateral decubitus, even during defecation or urination. Restraining no longer necessary for examination or treatment; moribund
**0**	Death	Death

Reproduced with permission from: Shores, A. **Development of a coma scale for dogs: Prognostic value in craniocerebral trauma**. <u>Proceedings 6th Annual Veterinary Medical Forum.</u> Washington, DC, pp 251-253, May 1988.

Appendix B – Performance status criteria (Modified Glasgow Coma Scale)

**Modified Glasgow Coma Scale (MGCS)**		
		**Score**
**Motor Activity**	Normal gait, normal spinal reflexes	6
	Hemiparesis, tetraparesis, or decerebrate rigidity	5
	Recumbent, intermittent extensor rigidity	4
	Recumbent, constant extensor rigidity	3
	Recumbent, constant extensor rigidity with opisthotonos	2
	Recumbent, hypotonia of muscles, depressed or absent spinal reflexes	1
**Brainstem Reflexes**	Normal pupillary light reflex and oculocephalic reflex	6
	Slow pupillary light reflex and normal to reduced oculocephalic reflex	5
	Bilateral unresponsive miosis with normal to reduced oculocephalic reflex	4
	Pinpoint pupils with reduced to absent oculocephalic reflex	3
	Unilateral, unresponsive mydriasis with reduced to absent oculocephalic reflex	2
	Bilateral, unresponsive mydriasis with reduced to absent oculocephalic reflex	1
**Level of Consciousness**	Occasional periods of alertness and responsive to environment	6
	Depression or delirium; capable of responding but response may be inappropriate	5
	Semicomatose; responsive to visual stimuli	4
	Semicomatose; responsive to auditory stimuli	3
	Semicomatose; responsive only to repeated noxious stimuli	2
	Comatose; unresponsive to repeated noxious stimuli	1
	**Total MGCS score**	**Scale**
	3-8	Grave
	9-14	Guarded
	15-18	Good

Reproduced with permission from: Shores, A. **Small animal coma scale revisited**. Proc 10th Ann Vet Med Forum, San Diego, May 1992, pp 749-750

Appendix C—Sample consent form for enrollment in the study, which dog owners will be provided and asked to review and sign.

CLIENT INFORMED CONSENT FORM

A Phase 1 Study of M032 (NSC 733972), a Genetically Engineered HSV-1 Expressing IL-12, in Canine Patients with Malignant Glial Brain Tumors

1. **Why is the study being performed?**

Some dogs are affected by brain cancer, particularly a brain tumor called a glioma, which is similar to brain tumors in humans. In both dogs and humans there are limited options for treatment of this tumor type, and the options available have not been shown to result in a long-term survival after tumor diagnosis. This study will help veterinarians determine if a modified virus (that has been shown to destroy cancer cells while leaving normal cells unharmed in humans) injected into gliomas in association with surgery will cause destruction of tumor and improve survival in dogs with gliomas.

2. **Which animals / patients can participate in the study?**

Initial enrollment in the study will occur if dogs have an MRI scan consistent with a glioma. Final enrollment will be determined by biopsy of the tumor at surgery.

3. **Why might my dog NOT be able to participate in the study?**

Dogs will be unable to participate in the study if they are pregnant, are undergoing treatment for other cancer and receiving chemotherapy, have a pacemaker, have a different disease affecting the brain, have a brain tumor that cannot be accessed surgically, do not have a brain tumor, do not have a glioma or have another disease that makes it unsafe to anesthetize them or treat them. Additionally, your dog cannot participate if you are unwilling to bring them back to the for **1)** recheck examinations at 14 days, then months 1, 2, 3, 4, 5, 6, 9, and 12, and study-funded concurrent MRI scans at 1, 3, and 6 months and **2)** at the time of euthanasia and necropsy (similar to human autopsy).

Finally, your dog cannot participate if you or anyone else in close contact with your dog have an underlying condition that compromises your immune system and makes it potentially unsafe for you to be around your dog after treatment. These conditions include pregnancy, diabetes mellitus, cancer for which you are receiving chemotherapy for or other diseases for which you are receiving drugs for that would lower your immune system defenses. **If you have any concerns about a condition you have or are unsure whether you have a condition which would make you at risk, we recommend you consult with your personal physician regarding your dog's enrollment in this study and your contact with your dog.** Although the virus used in this study has been shown to be ineffective at reproducing itself or causing infections outside of tumor tissue, it is unknown whether a compromised immune state could allow for infection. During the course of the study any blood from your dog could potentially carry the virus used in the study, and therefore this should be handled by wearing gloves and a facemask. The catheter that will be in your dog will be completely under the skin and therefore is not a risk to you for exposure. The study drug has been used successfully and safely in humans who are able to return home after treatment and who do not require isolation from their families or others.

4. **What will be happening to my animal if we participate in the study?**

If you participate in the study, your dog will undergo a physical examination, neurologic evaluation, routine tests to determine if they are healthy for general anesthesia (blood work, x rays of the chest, ECG). Your dog will then undergo general anesthesia so that an MRI of the brain may be performed. If the MRI confirms your dog has a brain tumor, your dog will be placed on a medication for seizures (levetiracetam). Your dog will then undergo general anesthesia for the purpose of performing surgery to remove as much of the tumor as possible, to obtain a biopsy, and to place very a small tube or tubes into the tumor to use later for administering virus to the tumor. Samples of your dog's tumor (biopsies) will be saved for evaluation by a pathologist to determine if your dog has a glioma. A CT scan of your dog will be performed after surgery to confirm the placement of tubes in the tumor site.

In addition to treatment with surgery, your dog may also be placed on medical therapy (prednisone) to decrease swelling associated with a brain tumor. After surgery, your dog will be recovered in our ICU hospitalization area. This is standard care for any dog undergoing brain surgery. 24-48 hours after surgery, once the results of your dog's biopsy are complete and confirm glioma, your dog will receive an infusion of cancer-destroying virus. This infusion will last 6 hours and will not require anesthesia. During the infusion your dog will be monitored for any negative effects of the drug so that infusion may be stopped if your dog is not tolerating it well.

If the biopsy from your dog is diagnosed as a tumor or disease other than glioma, your dog will not receive the treatment and at this point will be excluded from the study.

Your dog will be discharged from the hospital no sooner than 3 days after surgery. This time period may be longer if your dog shows signs that they need more supportive care from our medical team prior to discharge.

At each recheck examination your dog will have 5-10 mL blood drawn (between 1-2 teaspoons) for the purposes of monitoring their systemic health and their response to the virus administered. At the 1, 3, and 6 month rechecks your dog will undergo a second MRI examination to determine if residual brain tumor has changed in size. At the three month recheck your dog's tubes will be removed under sedation.

5. **Are there any benefits from the study for my animal?**

Yes, your animal will receive the commonly used therapy of surgery for the brain tumor. Additionally, your dog may benefit from the viral treatment as it may destroy any tumor cells left behind after surgery.

6. **Are there any risks or discomforts for my animal?**

The risks associated with this study are a reaction to the virus administered in the tumor area, a complication from surgery to remove the tumor or a complication from anesthesia. While your dog will be closely monitored for any signs of a problem, complications from the procedures and treatments may include: lethargy, decreased appetite, vomiting, seizures, pneumonia, hemorrhage (bleeding), infection, or death. These complications are the same as those expected with brain surgery for tumor removal alone. By enrolling your dog in this study, you acknowledge that you have been informed of these risks and are willing to proceed.

7. **Unforeseen adverse events**

Should an unforeseen adverse event associated with the study arise, your dog will be assessed immediately by our doctors to determine the best course of action. Treatment, if necessary, will be started immediately upon your consent. The study will provide up to $500 of supportive care required for treatment due to an unforeseen adverse event associated with the study.

8. **Are there treatment options other than participating in the study?**

There is not currently a "standard of care" for brain tumors in dogs that guides us as to a proven "best" choice of therapy (gold

standard). Should you not wish to participate in the study, we would still recommend an MRI to determine whether your dog has a brain tumor and, should they have one that can be treated with surgery, we would recommend surgery. Following surgery, in some cases, we recommend treatment with radiation therapy. Tumors that cannot be treated with surgery will often be considered for treatment with radiation therapy. Steroid therapy and drugs to prevent seizures (as described in this study) are routine aspects of therapy for dogs with this disease. There are also other experimental clinical trials available throughout the country for the treatment of gliomas in dogs.

9. **Owner responsibilities**

As the owner of the dog participating in the study, you agree to allow your dog to undergo blood sampling, screening diagnostic tests (chest x-rays, ECG), anesthesia for MRI scans, surgery, and treatment with virus. You agree to present your dog to the clinic for the purposes of these tests at your scheduled appointment times. Finally, in the difficult circumstance that you should choose to euthanize your dog or should your dog die for another reason, you agree to bring your dog to our hospital for euthanasia and for necropsy (similar to an autopsy in people).

10. **Participant dismissal**

Should your dog be determined to be an inappropriate candidate for anesthesia due to other health concerns, they will be dismissed from the study. Should your dog be diagnosed with a disease other than a brain tumor with the MRI scan, they will be dismissed from the study. Should your dog be diagnosed with a tumor other than glioma with a biopsy, they will be dismissed from the study. The study will still cover the cost of the surgery and initial MRI if a tumor other than glioma is diagnosed.

11. **Financial obligations of all parties**

The study will cover the cost of the initial diagnostics and MRI, surgery and treatment, recheck examinations, follow-up MRIs, and anesthetic events for your dog (this is approximately $7500 cost that the study covers for the care of your pet). The study does not cover the initial neurological evaluation ($85). Should you not comply with the follow up required for the study, you will be financially responsible for any and all charges associated with the care and treatment of your dog, including initial surgery and any follow up. The study will cover the cost of the necropsy and post mortem (after death) MRI should this need to be performed (this is approximately $600 cost that the study covers).

12. **Access to study information**

You will receive a report regarding the results of your dog’s MRI scans and any other diagnostic tests. The remainder of the information from this study will be put into an anonymous database and will not be available for review.

13. **Confidentiality**

The identity of you and your pet will remain confidential. If publication results from this study, identification methods will be used that prevent your pet’s true identity from being determined.

14. **Contact persons regarding this study**

[Facility specific information]

15. **Hospital review contact person**

[Facility specific information]

CLIENT INFORMED CONSENT FORM

16. **Owner acknowledgements:**

As legal owner of (or agent for) this animal, I understand and acknowledge the following:

a. **I am agreeing to participate in a research study at the [Facility]**

b. **I am free to withdraw my consent and to discontinue participation in the study at any time.**

c. **The decision for my animal to participate in this study is mine alone and *participation is voluntary.***

d. **The decision to withdraw from the study or to disregard the recommendations of the veterinarians involved in the study, relieves the investigators of current and future obligations (both medical and financial) to the pet and/or owner covered by this study.**

e. **Non-study treatment protocols have been discussed and I understand the relative benefits of those treatments.**

f. **I am responsible for decisions and financial obligations related to treatment(s) sought or required for my animal at __ or at any other veterinary facility that are NOT specified in this document. This would include treatment for disease progression or for medical complications - related or unrelated to the study.**

g. **If I withdraw my animal from the study, I may be responsible for medical, diagnostic and treatment costs incurred even if these were originally covered by the study.**

h. **Any decision to discontinue participation in this study, whether to pursue other therapies or to have my animal euthanized, does not entitle me to any financial reimbursement for costs incurred during participation.**

i. **I have discussed the procedures / benefits and risks of study participation and had the chance to have my questions about the study answered.**

Owner signature ____ Date ___________________

17. **Investigator assurances**

a. I have explained the study details and answered questions to the best of my ability.

b. If the animal is a patient in the [Facility], I have discussed enrolling the patient into this study with the senior faculty member responsible for this patient's care.

Primary Investigator _____________________________________ Date ___________________

Witness ____ Date ___________________

Adapted with permission from ***Client Informed Consent Form***; Amy B. Yanke, DVM, MS; Auburn University College of Veterinary Medicine

Appendix D—Possible Adverse Events Associated with M032 and Related International Medical Terminology (IMT) Terms

Category	Adverse Event	IMT Preferred Term
Allergy/Immunology	Allergic reaction/hypersensitivity	Hypersensitivity NOS
Blood/Bone Marrow	Leukocytes	Leucopenia NOS
Blood/Bone Marrow	Hemoglobin	Haemoglobin decreased
Blood/Bone Marrow	Hemolysis	Haemolysis NOS
Cardiovascular (General)	Edema	Oedema NOS
Cardiovascular (General)	Thrombosis/embolism	Thrombosis NOS
Constitutional Symptoms	Fatigue	Fatigue
Constitutional Symptoms	Fever	Pyrexia
Constitutional Symptoms	Weight loss	Weight decreased
Gastrointestinal	Nausea	Nausea
Hemorrhage	Hemorrhage/bleeding without grade 3 or 4 thrombocytopenia	Haemorrhage NOS
Hepatic	SGPT (ALT)	Alanine aminotransferase increased
Infection/Febrile Neutropenia	Infection without neutropenia	Infection NOS
Neurology	Pyramidal tract dysfunction	Upper motor neurone lesion
Neurology	Speech impairment	Speech disorder NEC
Neurology	Memory loss	Amnesia NEC
Neurology	Confusion	Confusion
Neurology	Mood alteration-depression	Depression NEC
Neurology	Personality/behavior	Personality change
Neurology	Neurology—other (specify, somnolence)	Not available
Neurology	Depressed level of consciousness	Depressed level of consciousness
Neurology	Seizure(s)	Convulsions NOS
Neurology	Neurology—other (specify, tumor progression)	Not available
Pain	Headache	Headache NOS

Note: The full list of IMT terms is available on the CTEP home page (http://ctep.info.nih.gov/CtepInformatics/IMT.htm).

Appendix E—Possible Adverse Events Associated with Indoximod and Related International Medical Terminology (IMT) Terms

**Category**	**Adverse Event**	**IMT Preferred Term**
Allergy/Immunology	Allergic reaction/hypersensitivity (including drug fever)	Hypersensitivity NOS
Blood/Bone Marrow	Hemoglobin	Haemoglobin decreased
Blood/Bone Marrow	Lymphopenia	Lymphopenia
Blood/Bone Marrow	Neutrophils/granulocytes (ANC/AGC)	Neutropenia
Blood/Bone Marrow	Platelets	Platelet count decreased
Cardiovascular (General)	Edema	Oedema NOS
Constitutional Symptoms	Fatigue	Fatigue
Gastrointestinal	Anorexia	Anorexia
Gastrointestinal	Constipation	Constipation
Gastrointestinal	Dehydration	Dehydration
Gastrointestinal	Diarrhea (without colostomy)	Diarrhoea NOS
Gastrointestinal	Nausea	Nausea
Gastrointestinal	Vomiting	Vomiting NOS
Hepatic	Alkaline phosphatase	Blood alkaline phosphatase NOS increased
Metabolic/Laboratory	Hyperglycemia	Hyperglycaemia NOS
Metabolic/Laboratory	Hyponatremia	Hyponatremia
Neurology	Neuropathy-sensory	Peripheral sensory neuropathy
Pain	Abdominal pain or cramping	Abdominal pain NOS
Pain	Arthralgia (joint pain)	Arthralgia
Pain	Headache	Headache NOS
Pain	Pain-other (specify, back pain)	Not available
Pulmonary	Dyspnea (shortness of breath)	Dyspnoea NOS
Pulmonary	Cough	Cough
Renal/Genitourinary	Creatinine	Blood creatinine increased

Note: The full list of IMT terms is available on the CTEP home page (http://ctep.info.nih.gov/CtepInformatics/IMT.htm).
