# Supplementary material for: The One Health Consortium: Design of a Phase I Clinical Trial to Evaluate M032, a Genetically Engineered HSV-1 Expressing IL-12, in Combination With a Checkpoint Inhibitor in Canine Patients With Sporadic High Grade Gliomas
Source: Front Surg. 2020 Aug 28;7:59. doi: 10.3389/fsurg.2020.00059 (PMC7484881; doi:10.3389/fsurg.2020.00059)
Supplement: Supplementary file 2 [file Table_2.docx]

| **Abbreviations** | |
| --- | --- |
| ALP | Alkaline phosphatase |
| ALT | Alanine aminotransferase |
| AST | Aspartate aminotransferase |
| BUN | Blood urea nitrogen |
| CNS | Central Nervous System |
| GBM | Glioblastoma multiforme |
| CpG | Cytosine triphosphate deoxynucleotide phosphodiester-linked to guanine triphosphate deoxynucleotide; recognized by Toll Receptor 9 |
| HSV | Herpes Simplex Virus |
| oHSV | Oncolytic HSV |
| M002 | Mutant γ_1_ 34.5-deleted oHSV engineered to express mouse Interleukin-12 |
| M032 | Mutant γ_1_ 34.5-deleted oHSV engineered to express human Interleukin-12 |
| G207 | Doubly mutant oHSV; both copies of γ_1_ 34.5 gene have been deleted and virus ribonucleotide reductase (U_L_39) has been insertionally inactivated. |
| IDO | Indoleamine 2,3-dioxygenase |
| AdV | adenovirus |
| AAV | adeno-associated virus |
| TDO | tryptophan dioxygenase |
| Tregs | regulatory T cells |
| MDSCs | myeloid derived suppressor cells |
| MTD | Maximum Tolerated Dose [In North America, the Maximum Tolerated Dose (MTD) is the RP2D; elsewhere, the MTD is considered the dose level above the RP2D] |
| RP2D | Recommended Phase 2 Dose (usually the highest dose with acceptable toxicity, defined as the dose level producing around 20% of dose-limiting toxicity) |
| SGOT | Serum glutamic-oxaloacetic transaminase (AST) |
| NOAEL | No-Observed-Adverse-Events-Level |
| PCR | Polymerase chain reaction |
| MKPS | Modified Karnofsky Performance Status |
| DLT | Dose-limiting toxicity |
| GMP | Good Manufacturing Practice |
| CFSE | Carboxyfluorescein succinimidyl ester |
| FACS | Fluorescence-activated cell sorting flow cytometry |
| IACUC | Institutional Animal Care and Use Committee |
| IBS | Institutional Biosafety Committee |
| ADCC | antibody-dependent cellular cytotoxicity |
| NK cell | natural killer cell |
| CD3 | Cluster of Differentiation antigen 3 – expressed on mature T lymphocytes |
| CD4 | Cluster of Differentiation antigen 4 – expressed on helper T lymphocytes |
| CD8 | Cluster of Differentiation antigen 8 – expressed on cytotoxic T lymphocytes |
| CD56 | Cluster of Differentiation antigen 56 – expressed on activated CD8+ T and γδ T lymphocytes and dendritic cells |
| CD25 | Cluster of Differentiation antigen 25 – Interleukin 2 receptor alpha expressed on activated T and B lymphocytes, myeloid precursors and resting memory T cells |
| FoxP3 | Forkhead Box Protein 3 expressed on natural and adaptive/induced T regulatory cells |
| CD79a | Cluster of Differentiation antigen 79A – B cell receptor-associated alpha chain |
| CD21 | Cluster of Differentiation antigen 21 – B cell complement receptor type 2 |
| CD11 | Cluster of Differentiation antigen 11 – integrin alpha component |
| CD44 | Cluster of Differentiation antigen 44 – cell-surface glycoprotein on many cell types involved in cell-cell interaction, migration, cell adhesion |
| CD45RA | Cluster of Differentiation antigen 45RA- Protein tyrosine phosphatase receptor C, leukocyte common antigen |
| CD62L | Cluster of Differentiation antigen 62L – L-selectin, a cell adhesion molecule found on lymphocytes; important in lymphocyte-endothelial interactions |
